# Protective Effects of 1,8-Cineole Microcapsules Against Inflammation and Gut Microbiota Imbalance Associated Weight Loss Induced by Heat Stress in Broiler Chicken

**DOI:** 10.3389/fphar.2020.585945

**Published:** 2021-01-14

**Authors:** Zhihui Jiang, Maojun Luo, Wentao Ma, Shengming Ma, Yao Wang, Kunpeng Zhang

**Affiliations:** ^1^ Henan Joint International Research Laboratory of Veterinary Biologics Research and Application, Anyang Institute of Technology, Anyang, China; ^2^ College of Food Science and Technology, Henan Agricultural University, Zhengzhou, China

**Keywords:** microcapsules, gut microbiota, inflammation, heat stress, 1,8-cineole

## Abstract

Intestinal microbiota dysregulation is considered the primary trigger of low-grade inflammation responsible for weight loss due to heat stress. 1,8-Cineole is the major bacteriostatic agent in eucalypt and possesses remarkable anti-inflammatory properties. However, the mechanisms of its effect on intestinal microbiota remain unclear. In this study, 1,8-cineole was prepared into microcapsules prior to use as feed supplement in chickens. The microencapsulation efficiency and chemical stability of 1,8-cineole microcapsules were evaluated. The chicken treatment with 1,8-cineole microcapsules (1 or 3%) for 45 days, in the presence or absence of heat stress for fifteen days, commenced on Day 31, with or without an antibiotics mix (Abx) for three days on Day 27. Performance parameters were measured once a week from Day 30 through Day 45. Surface and entrapped concentration of 1,8-cineole was estimated as 7.89 g/100 g powder in the microcapsules. The time to maximal concentration (T_max)_, terminal half-life (T_1/2_), and the area under plasma concentration-time curve (AUC_0-t_) of the encapsulated 1,8-cineole were higher than those of the nonencapsulated in treated chickens, although the maximal concentrations (C_max_) were similar. Chickens treated under higher temperatures with 1,8-cineole microcapsules exhibited lower levels of grade inflammation and higher body weight gain. Dietary 1,8-cineole microcapsules recovered the normal structure of upper ileum and altered the ratio of gut microbiota under heat stress and increased the ratio of *Lactobacillus* and *Escherichia*, whereas the proportion of *Salmonella* decreased based on 16S rRNA analysis of the upper ileum microbiota. *In vitro*, 1,8-cineole effectively inhibited the growth of *Salmonella* as demonstrated by inhibition zone assay. In summary, our findings elucidated the interaction between 1,8-cineole and intestinal microbiota as a new mechanism for the anti-heat stress effect of 1,8-cineole in preventing low-grade inflammation and weight loss. The results suggest that 1,8-cineole microcapsules may be a good feed supplement to protect against heat stress injury.

## Introduction

The poultry industry ranks first among other livestock industries worldwide. Poultry farming systems are affected by a variety of climatic factors. Among these factors, environmental pressure has become a focus due to increase in environmental pollution and public awareness (Cedraz De Oliveira et al., 2017; Farag and Alagawany, 2018). Rising temperature affects the sensitivity of chickens to bacteria and parasites in the environment (Alhenaky et al., 2017; Tsiouris et al., 2018). Heat stress poses a serious threat to homeostasis and has potential adverse effects on overall health, growth performance, intestinal morphology, physiology, and immunity of poultry (Alhenaky et al., 2017; Volodina et al., 2017; Nawab et al., 2018). Food safety issues related to heat stress are of special significance due to an abundance of available scientific information and public awareness.

Heat stress can disorganize the distribution and proportion of intestinal microbiota (He et al., 2019), and antibiotics are often used to boost intestinal immunity by breeders. However, in Europe in 2006 and China in 2020, the addition of low dose antibiotics as growth-promoting agents to livestock feed was banned, accelerating research into suitable natural substitutes with similar beneficial effects. Among these substitutes, plant feed additives are promising but diverse additives that promote the health and performance of poultry (Windisch et al., 2008; Hashemi and Davoodi, 2010). Feed palatability and high ileum digestibility of feed are considered the main mechanisms of the growth-promoting effects of plant-derived feed additives, most of which increase the secretion of islet enzymes (Yi et al., 2018). In recent studies, effects of these additives on intestinal microbiota indicate their beneficial effect on performance (Zhai et al., 2018), antioxidative protection systems (Chowdhury et al., 2018), the immune system (Yang et al., 2018), and anti-inflammatory responses of animals (Griss et al., 2018; Liu et al., 2018).

Essential oil from food or medicine is one of the phytogenic feed additives. It has great potential on the livestock breeding and is generally considered to be less toxic, being natural and residues-free compared with exogenous synthetic compounds. Essential oils are considered growth promoters in poultry feed (Zhang et al., 2014). Previous studies showed that the essential oils, eugenol, thymol, and carvacrol, had powerful antibacterial activity against *Salmonella typhimurium*, *Escherichia coli*, and other pathogenic bacteria (Marchese et al., 2017; Hu et al., 2018; Park et al., 2018). 1,8-Cineole (eucalyptol) is the main component of many plant essential oils, mainly extracted from *Eucalyptus globulus* oil (Merghni et al., 2018). Because of its pleasant taste and aroma, 1,8-cineole is often used in food, perfumes, and cosmetics (Poitou et al., 2017). The pure 1,8-cineole is used to treat respiratory infections, such as bronchitis or common cold, and as an additional treatment for sinusitis (Yang et al., 2010; Greiner et al., 2013). Clinical experiments have confirmed that 1,8-cineole has antimicrobial and antiseptic properties *in vitro* (Kifer et al., 2016; Simsek and Duman, 2017). In this study, we investigated possible molecular mechanisms employed by 1,8-cineole microcapsule to mediate its effects on the growth performance and intestinal microbiota of chickens under heat stress with or without antibiotics.

## Materials and Methods

### Preparation of Emulsions and Microcapsules and Spray-Drying

The aqueous phase was prepared by dissolving hydroxypropyl methyl cellulose (HPMC) and maltodextrin (MD) at 45°C and 12 h after magnetic stirring. Colloidal silicon (CSD) was dispersed into the solution. 1,8-Cineole was dissolved in neutralizing olive oil (30% w/w) employed as the lipid core (C). HPMC, MD, and CSD were used as well material (WM). To prepare the emulsion, the lipid core was added to the aqueous phase using an automixer homogenizer at 12,000 rpm for 6 min and immediately spray-dried. After the different conditions were tested, the optimization condition was as follows: the WM:C ratio was prepared (2:1) and ratio of W component was 1:0.5:0.5 (HPMC:MD:CSD). The sample was vacuum freeze-dried. Prefreezing temperature was 80°C for 6 h and freezing temperature was −40°C for 24 h at 10 Pa. The powder was stored at 4°C drying condition for subsequent use.

### Characterization of Microcapsules

#### Microencapsulation Efficiency

Microencapsulation efficiency (ME) was defined as the amount of 1,8-cineole loaded in the microparticles. Microcapsules (20 g) were placed in an extraction tube and covered with cotton. The powder was extracted for 1.5 h with n-hexane and evaporated to dry at 30°C. The weight of the oil retained (i.e., extracted from the microcapsules) in the Soxtec cups was calculated. The amount of entrapped oil was analyzed by subtracting the surface oil from the total oil. The ME was assessed as the percentage of entrapped oil to the total amount of encapsulated oil.

#### Chemical Stability of Microcapsules During Storage

The chemical stability of 1,8-cineole in the microencapsulation was assessed during storage for 4, 6, 8, 10 weeks at 20 and 35°C. The surface of each microcapsule was cleaned with absolute ethanol and 1 g washed powder was mixed in a centrifuge tube with 5 ml distilled water and 5 ml *n*-hexane. The mixture was stirred in a centrifuge tube with *n*-hexane, blended on a rotary mixer for 5 min, and ultrasonicated for 30 min. The *n*-hexane in the mixture was separated by centrifugation at 5,000 × g for 5 min. The content of 1,8-cineole was measured in *n*-hexane by GC-MS (Agilent, California, United States), equipped with a nonpolar HP-5 column (Sigma-Aldrich, United States). The flow rate was 3 ml/min, with a split ratio of 15:1. The column temperature was controlled at 40°C for 2 min and then heated from 40 to 120°C at a rate of 5°C/min. The temperature was maintained at 120°C for 5 min and increased from 120 to 180°C at a rate of 10°C/min.

The mass spectrometry analysis conditions are as follows: the electron ionization (EI) ion source, electron energy 70 eV, quadruples temperature 180°C, electromagnetic voltage 2165 V, interface temperature 250°C, solvent delay time 3 min, and *m/z* 40–550 amu. The percentage of 1,8-cineole residue was calculated as follows:

$$\frac{\text{Amount }\left(\text{g}\right)\;\text{measured at storage time }\left(\text{tx}\right)\;}{\text{Amount }\left(\text{g}\right)\text{ measured at zero storage time }\left(\text{t}0\right)}\times 100\text{\%}.$$

Semilog curves of 1,8-cineole retention over storage time were calculated. The slope of the stability curve was determined by regression analysis, and the half-life (t_1/2_) was calculated as 0.693/*k* base on the slope (*k*) of the curve (Krishnan et al., 2005).

#### Pharmacokinetic Parameters of 1,8-Cineole Microcapsules in Plasma

Ten 30-day-old Ebayka broilers were randomly divided into two groups, five in each group (n = 5). The 1,8-cineole group were given 1 ml/kg (10% 1,8-cineole dilution by olive oil), and 1,8-cineole microcapsules group received 1 mg/kg. Prior to feeding with the 1,8-cineole (1 ml/kg, 10%) or 1,8-cineole microcapsules (1 mg/kg), the chickens were subjected to a fast for 12 h with free access to water. Blood samples (0.2 ml) were obtained from the wing vein of the same chickens at 0.2, 0.5, 0.75, 1, 1.5, 2, 4, 6, 8, and 10 h after dosing. The samples were collected into EP tube precoated with heparin in a process that took 1 h. The samples were centrifuged and the separated plasma was removed and stored at 4°C until analysis. Ten microliters of the filtered sample liquid was injected into a gas chromatograph (GC, Agilent 7890B, California, United States) system for analysis. The standard curve consisted of a sample containing 10, 20, 40, 80, 160, and 320 ng/ml of the 1,8-cineole. Plasma quality control samples spiked with 20 ng/ml (low), 80 ng/ml (medium), and 320 ng/ml (high) of the 1,8-cineole were accordingly prepared to measure the accuracy and precision of the method.

Chromatography was performed with an GC system with capillary column HP-5 (30 m × 0.32 mm, 0.25 μm particles). Flame ionization detector (FID) temperature was 250°C and injector temperature was 240°C. The column temperature was started at 50°C, increased to 75°C at 5°C/min, maintained for 6 min, and then increased to 200°C at 20°C/min and maintained at 3 min. The data of 1,8-cineole content was analyzed using DAS 2.1 for 1,8-cineole pharmacokinetic parameters, such as the time to maximal concentration (T_max_), maximal concentration (C_max_), elimination half-life (t_1/2_), the area under plasma concentration-time curve (AUC_0-t_), and the area under the plasma concentration-time curve to time infinity (AUC_0-∞_).

### Animals and Treatments

The one-day-old Ebayka broilers were treated in a poultry farm from Puyang Dexin Food Co., Ltd. During the experiments, birds were allowed to freely feed on corn and soybean-based diets and water. The chicken population density was 12 chickens/m^2^.

**FIGURE 1:**
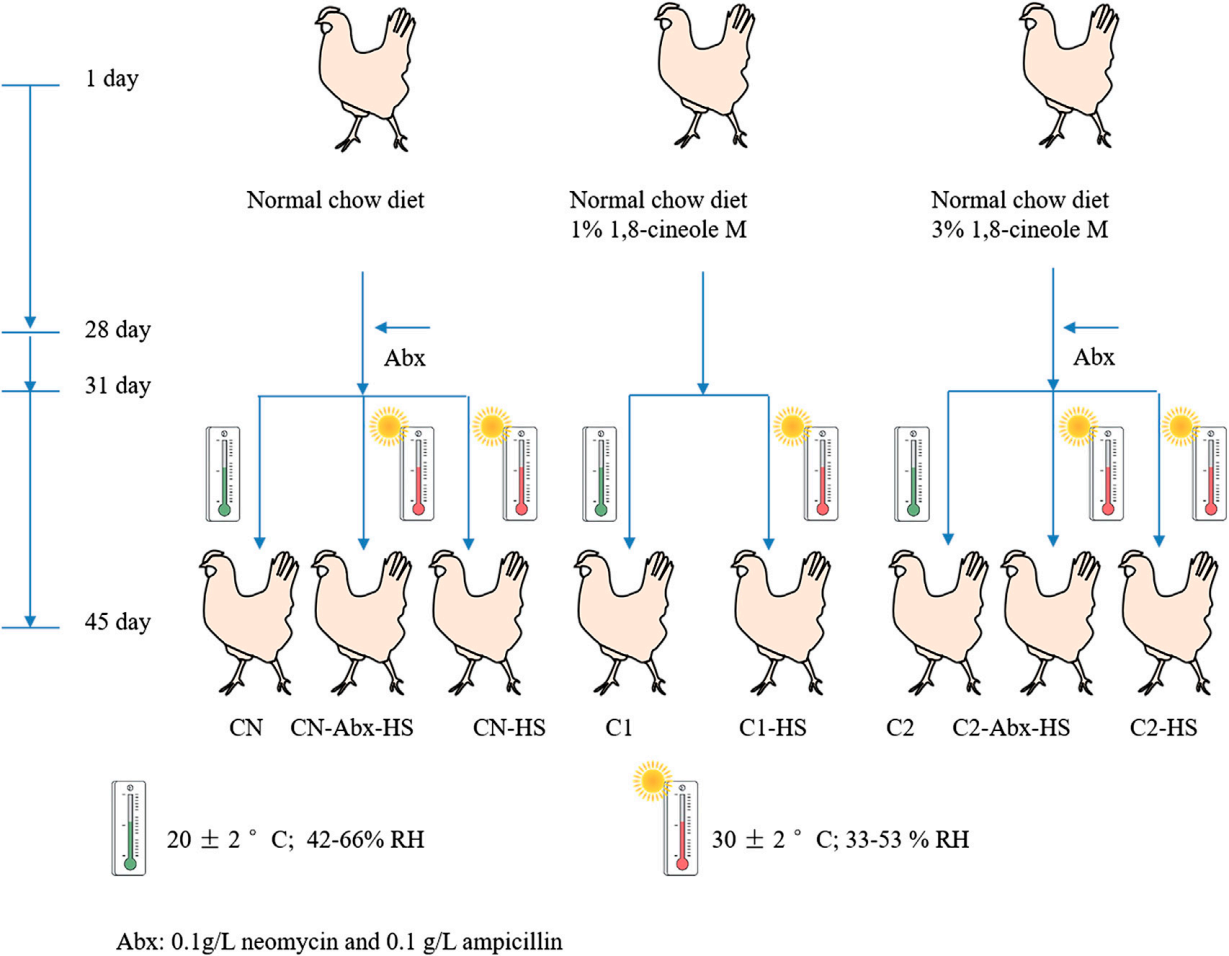
Flow chart of chicken processing. Notes: The chickens were separated into eight groups with twenty chickens per group for observation of performance parameters. Six of the chickens from each testing group were subjected to molecular biological index testing. CN: normal chow-diet for forty-five days; CN-HS: normal chow-diet for forty-five days and heat stress treatment on Day 31 of experiment for fifteen days; C1: 1% 1,8-cineole microcapsules for forty-five days; C1-HS: 1% cineole microcapsules for forty-five days and heat stress treatment on Day 31 of experiment for fifteen days; C2: 3% 1,8-cineole microcapsules for forty-five days; C2-HS: 3% 1,8-cineole microcapsules for forty-five days and heat stress treatment on Day 31 of experiment for fifteen days; CN-Abx-HS: normal chow-diet for forty-five days and Abx for three days on Day 28 and HS treatment on Day 31 for fifteen days. C2-Abx-HS: 3% 1,8-cineole microcapsules for forty-five days and Abx for three days on Day 28 and HS treatment on Day 31 for fifteen days.

The chickens were divided into three groups. One group was conventionally raised with normal chow-diet (CN), and the other two groups were fed with 1% or 3% 1,8-cineole microcapsules in the normal chow-diet (C1 and C2, respectively). On Day 31, half of each group was subjected to non-heat stress treatment by being kept at environmental conditions of 20 ± 2°C, 42–66% relative humidity. Others were subjected to chronic heat stress (HS) treatment (CN-HS, C1-HS and C2-HS) by being placed in an environmental control room and continuously exposed to a high temperature of 30 ± 2°C and relative humidity of 33–53%, 24 h per day for 15 successive days. To investigate the role of gut microbiome on host metabolism, one-half of the CN-HS and C2-HS groups were treated with antibiotics mix (Abx, 0.1 g/L neomycin and 0.1 g/L ampicillin) in drinking water on Day 28 for three consecutive days (CN-Abx-HS, C2-Abx-HS). Eight treatment groups and twenty chickens in each treatment group were observed for performance parameters; six of these chickens were selected for molecular biological index testing. The processing flow chart is shown in Figure 1.

### Performance Parameters

Body weight and total week feed intake/replicate were measured once a week from Day 30 to Day 45. Mortality and illness (drooping spirit, diarrhea, and tumbled feathers) were recorded daily, and feed conversion rates were revised based on the number of birds removed for sampling.

### Observation of Intestinal Tissue

Upper ileum of chickens from all each group were separated and fixed in 10% neutral buffered formaldehyde solution for one week. For staining, 5-µm serial sections were obtained after the tissues were embedded in 60°C paraffin. Slides were stained with hematoxylin and eosin (H & E) and examined under an optical microscope (Leica DMi8, Germany).

### Upper Ileum Microbiota Characterization Using 16S rRNA Gene Amplification and Sequencing

#### PacBio Sequencing

Total DNA was prepared from upper ileum contents for 16S rRNA gene targeted sequencing and bacterial community profiling. The 16S rRNA from upper ileum was analyzed by BGI company (BGI. Co. Ltd., Shenzhen, China). The samples were used to construct the library. Using barcode label, the full-length amplification primer amplified the 16S region. To repair damaged amplicon, the end was connected with the known connector; the enzyme reaction was eliminated. After BluePippin sorting, a dumbbell-shaped library was obtained. After the library was tested by Agilent 2,100 and Qubit HS, the data was analyzed, and result interpretation was performed using PacBio sequencing.

#### Operational Taxonomic Unit Clustering

Operational taxonomic unit (OTU) clustering of ccs reads for quality tailoring of each environmental sample was done using WSEARCH (-cluster_size, -strand both, -id 0.97,-sizeout). Add “; size = <read_quality>” before clustering. This ensured that the –cluster_size algorithm was from the highest to lowest read quality before the cluster and that the highest read quality became the center of mass of OTU.

#### Statistical Analysis

The beta diversity analysis included the principal component analysis (PCA) and principal coordinate analysis (PCoA), calculated by weighted Unifrac and unweighted Unifrac, respectively. The abundance of taxonomic composition of bacteria was observed by R method. The significance analysis was performed using one-way ANOVA.

### Determination of Antimicrobial Characteristics of 1,8-Cineole

Antimicrobial activity of 1,8-cineole was assessed by inhibition zone (IZ) assay and minimum inhibitory concentration (MIC) assay. Pathogenic bacteria *E. coli* (isolated from human or chicken), *Staphylococcus aureus*, and *Bacillus subtilis* were obtained from a microbiology lab (Anyang Institute of Technology). The multidrug resistant *E. coli* (K88) and *S. aureus* (MRSA; methicillin-resistant *S. aureus*) were obtained from State Key Lab of Microbial Resources (The Institute of Microbiology CAS) and *Salmonella* (CUCC542) was obtained from microbiology lab (Northwest Agriculture & Forestry University).

To assess the effect of 1,8-cineole against bacterial activity based on IZ, bacteria were cultured as suspension colonies overnight in lysogeny broth at 37°C. Culture was adjusted to 1 × 10^8^ CFU·ml^−1^ and plated on LB agar. Sterile discs (6 mm in diameter) were saturated with 1,8-cineole and placed on inoculated bacteria growth. The setup was incubated at 37°C for 1 day. The diameter of the IZ was measured.

To determine its MIC, 1,8-cineole was diluted serially from 1,000 μL/ml to 15.5 μL/ml using a selective broth. A 100 μL aliquot was pipetted into each well of a 96-well plate containing 90 μL broth and 10 μL of working inoculum suspension (1 × 10^4^ CFU/ml). The negative control wells contained no 1,8-cineole. The plates were incubated for 24 h at 37°C, followed by the addition of 40 μL of p-iodonitrotetrazolium purple solution (INT, Sigma-Aldrich) to each well with further incubation for 5 h. The lowest dilution with no discoloration was considered the MIC value of 1,8-cineole. The tests were repeated ten times.

### Quantitative real-time PCR Analysis of Inflammation-Related Genes in Upper Ileum Tissues

Total mRNA was isolated from frozen upper ileum tissues using a commercial Total RNA kit. cDNA was amplified by using 2 × SYBR Green I PCR Master Mix using quantitative real-time PCR (qPCR) (Vazyme, Nanjing, China). The PCR procedure was performed under the following conditions: 95°C for 30 s, followed by 30 cycles of 95°C for 15 s, 58°C for 30 s and 72°C for 30 s. The PCR primers used were as follows: TNF-α, Up: GCC​CTT​CCT​GAT​ACC​AGA​TG, Low: ACACGACAGCCAAGTCAA CG, 98 bp; IL-1β, Up: CAG​CAG​CCT​CAG​CGA​AGA​G, Low: CTGTGGTGTGC TCAGAATCCA, 86 bp; IL-6, Up: AAA​TCC​CTC​CTC​GCC​AAT​CT, Low: CCCT CAC​GGT​CTT​CTC​CAT​AAA, 106 bp; IL-10, Up: CGC​TGT​CAC​CGC​TTC​TTC​A, Low: TCCC GTT​CTC​ATC​CAT​CTT​CTC, NF-қB, Up: TCA​ACG​CAG​GAC​CTA​AAG​ACA​T, Low: GCA​GAT​AGC​CAA​GTT​CAG​GAT​G, 162 bp; β-actin, Up: CCGCTCTATGA AGGCTACGC, Low: CTC​TCG​GCT​GTG​GTG​GTG​AA, 128 bp. Primer specificity and product purity were inferred from the dissociation and melting curve. Relative abundance of each mRNA was calculated with the formula 2−^(ΔΔCt)^.

### Statistical Analyses

GraphPad Prism 5.0 was used for statistical analysis (GraphPad Software, Inc., San Diego, CA). Two groups of experiments were analyzed using Student’s *t* tests, and more than two groups were analyzed using one-way analysis of variance with post hoc Bonferroni’s multiple-comparison tests. PCA was used for the correlation between groups and results. Statistical Product and Service Solutions (SPSS, IBM, Chicago, United States) was used to analyze the significant difference among each treatment group.

## Results

### Comparison of 1,8-Cineole Microcapsules and 1,8-Cineole Stability and Pharmacokinetics

**FIGURE 2:**
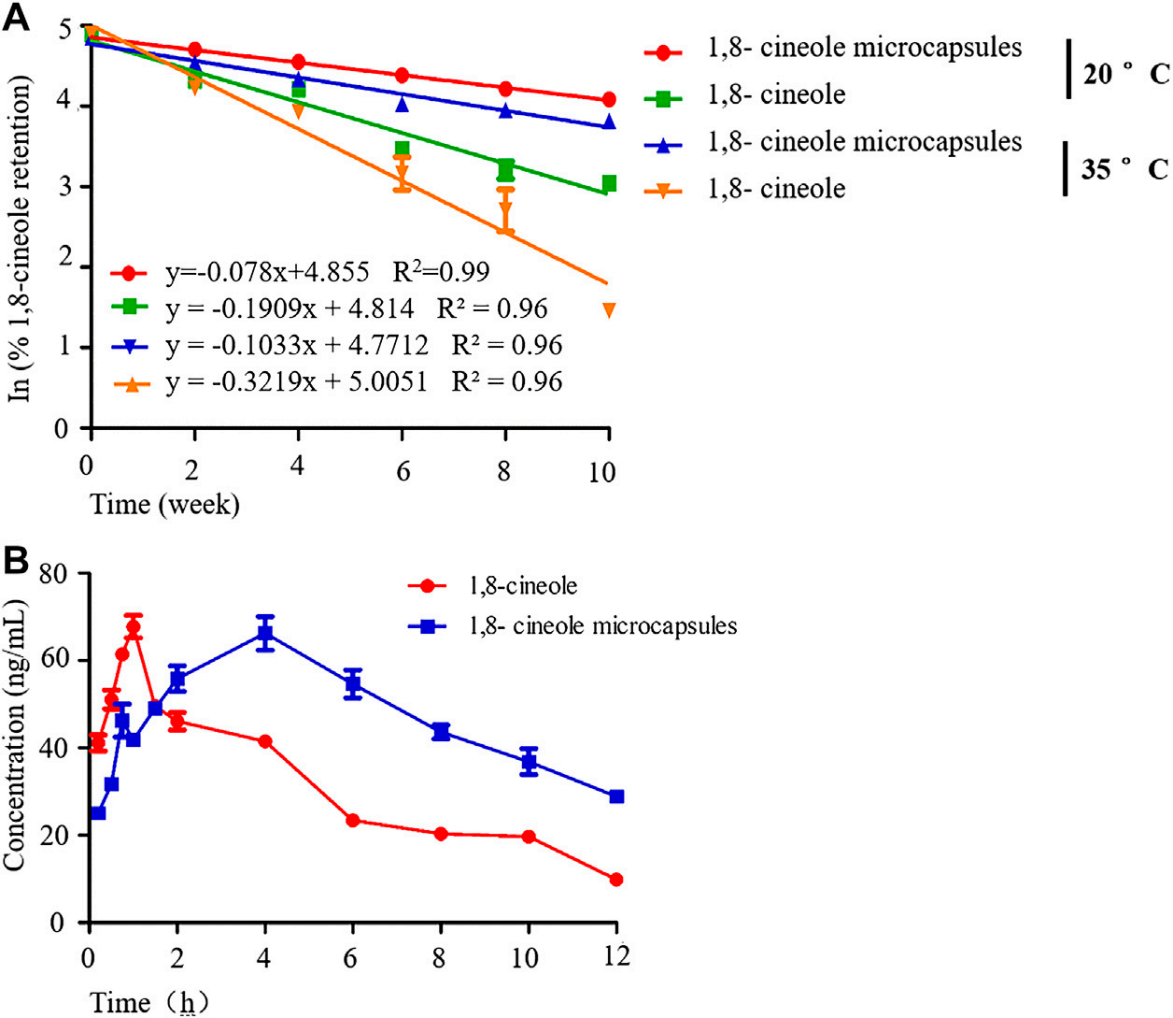
The stability and pharmacokinetics of 1,8-cineole microcapsule. Notes: **(A)** the stability of 1,8-cineole microcapsule and 1,8-cineole at 20 and 35°C; **(B)** the concentration of 1,8-cineole in plasma after 1,8-cineole microcapsule or 1,8-cineole treatment at the indicated times; n = 5.

Surface and entrapped 1,8-cineole was 7.89 g/100 g powder and its ME was 7.89%. The regression analysis of the concentrations of 1,8-cineole in the microcapsule after 10 weeks of storage at 20 and 35°C is shown in Figure 2A. The correlation coefficients (*R* ^2^) for the two curves are 0.99 and 0.96, respectively. The linear change in the retention of 1,8-cineole with storage time in the semilog plot indicated that the loss of 1,8-cineole followed first-order kinetics; the loss rate decreased constantly with increasing storage time. The microcapsules were better at retaining 1,8-cineole than the nonencapsulated control. The t_1/2_ of 1,8- cineole was longer (25.75 weeks) in microcapsules than in the nonencapsulated (11.04 weeks) at 20°C. However, higher loss of 1,8-cineole was observed at 35°C storage (16.05 weeks). The *k* value of 1,8-cineole in microcapsules and the nonencapsulated was 0.027 and 0.065, respectively, at 20°C and 0.043 and 0.099, respectively, at 35°C. This was expected given the higher gas permeability of the polymer wall and oxidation reactions of 1,8-cineole occur at higher temperatures (Kuang et al., 2010).

As seen in Table 1 and Figure 2B, the ADME of 1,8-cineole microcapsule and 1,8-cineole in chicken were different. The AUC_0-t_ was significantly increased after encapsulation, unlike in the nonencapsulated treatment (Figure 2B; Table 1). The achieved C_max_ of 1,8-cineole and 1,8-cineole microcapsules was 67.71 and 65.74 ng/ml, respectively (Table 1). Absorption in the encapsulated treatment was slower with a T_max_ of 4 h, compared to the T_max_ of 1 h in the nonencapsulated treatment. The t_1/2_ of the encapsulated (5.75 h) was longer than that of the nonencapsulated (2.08 h).

**TABLE 1:** Pharmacokinetic parameters of 1,8-cineole in chicken after oral administration of 1,8-cineole and 1,8-cineole microcapsules.

Treatment	C_max_ (ng/ml)	T_max_ (h)	T_1/2_ (h)	AUC_0-t_ (ng h/ml)	AUC_0-∞_ (ng h/ml)
1,8-Cineole	67.7 ± 5.45	1.0 ± 0.45	2.1 ± 0.28	153.6 ± 5.38	184.3 ± 6.49
1,8-Cineole microcapsules	65.7 ± 2.51	4.0 ± 0.20^a^	5.8 ± 0.75^a^	204.5 ± 10.83^a^	248.9 ± 24.51^a^

^a^ There was significant difference between 1,8-cineole and 1,8-cineole microcapsules treatment (*p* < 0.05); n = 5.

### Dietary 1,8-Cineole Microcapsules Increase Weight Gain and Reduce Low-Grade Inflammation

**FIGURE 3:**
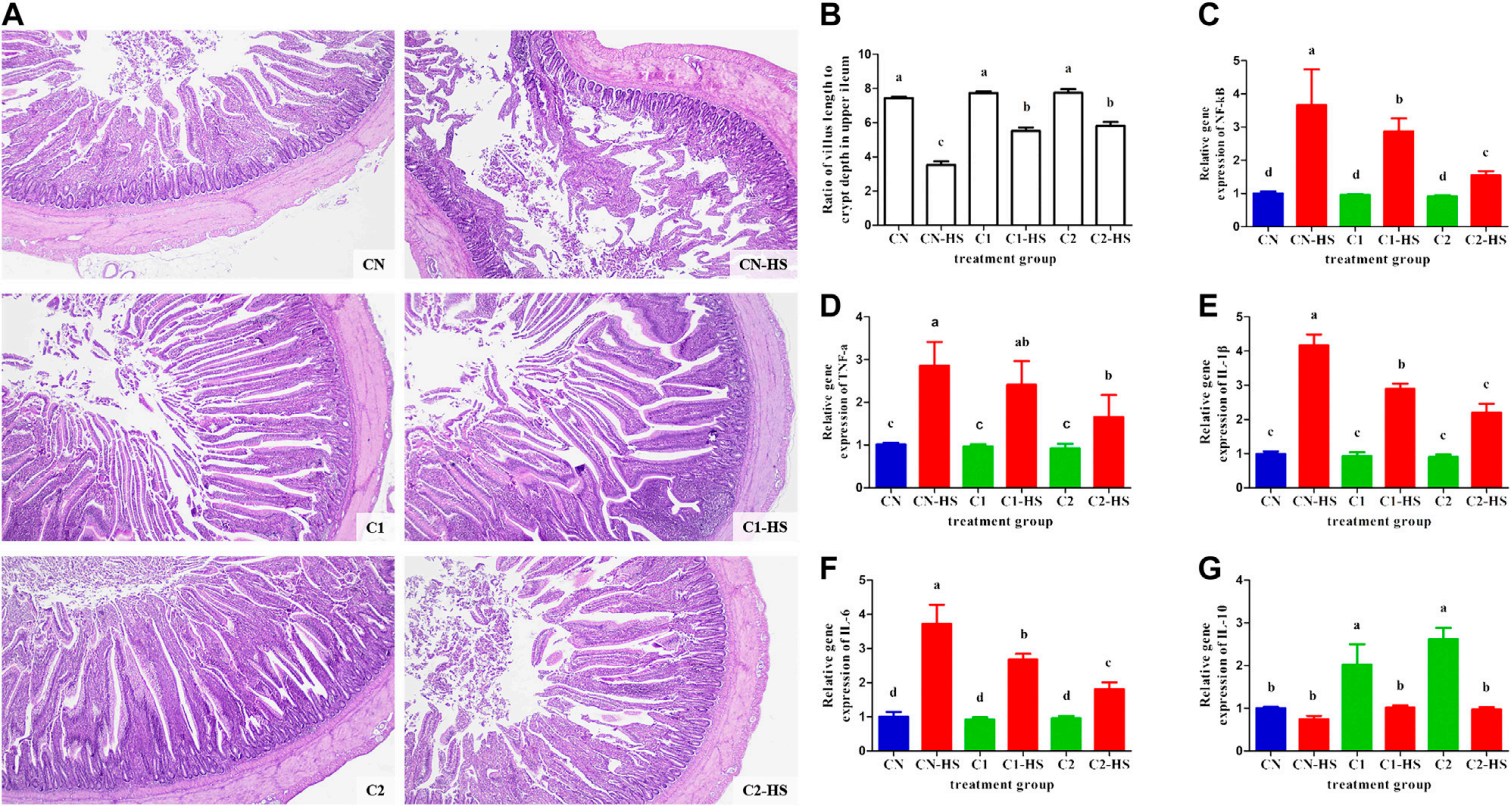
Effect of 1,8-cineole microcapsule on pathological change and inflammation expression in upper ileum. Notes: **(A)** pathological changes in the upper ileum; **(B)** ratio of villus length to crypt depth; **(C**–**G)** the NF-қB, TNF-α, IL-1β, IL-6, and IL-10 gene expression. n = 6.

To investigate the impact of 1,8-cineole microcapsules on weight and performance parameters under heat stress, chickens were fed a normal diet with or without 1,8-cineole microcapsule (1% or 3%) supplementation with or without heat stress. As shown in Table 2, compared with CN group, dietary 1,8-cineole microcapsules significantly increased the chicken weight with 118.5 g (C1) and 212.2 g (C2) after feeding 30 days 1% and 3% 1,8-cineole. As expected, heat stress induced the decline of the growth parameters (body weight, average daily gain, and average daily feed intake) in the CN-HS group, increasing mortality and illness. However, ingestion of 1% or 3% 1,8-cineole microcapsule significantly prevented these heat stress induced phenotypes in the C1-HS and C2-HS groups. Assessment of intestinal tissue showed that 1,8-cineole microcapsules increased heat stress induced decrease in the ratio of villus and depth (Figures 3A,B). Furthermore, markers of consumption index (feed conversion ratio, mortality, and illness) were significantly decreased by 1,8-cineole microcapsules in C1-HS and C2-HS groups, compared with CN-HS group (Table 2).

**TABLE 2:** Effect of chronic heat stress on chicken performance.

Parameters	Treatments							
	CN	CN-HS	C1	C1-HS	C2	C2-HS	CN-Abx-HS	C2-Abx-HS
Initial body weight (g), Day 0*	1,324.2 ± 26.84^c^	1,355.3 ± 23.45^c^	1,442.7 ± 18.94^b^	1,422.6 ± 15.38^b^	1,536.4 ± 19.87^a^	1,531.9 ± 21.85^a^	1,349.3 ± 21.62^c^	1,501.4 ± 32.53^a^
Initial body weight (g), Day 7*	1753.7 ± 23.56^c^	1,536.8 ± 26.91^d^	1955.8 ± 21.85^b^	1723.4 ± 20.81^c^	2035.8 ± 17.42^a^	1733.3 ± 25.62^c^	1802.4 ± 40.22^c^	1748.7 ± 55.22^c^
Initial body weight (g), Day 14*	2,170.5 ± 19.31^d^	1825.2 ± 25.79^e^	2,454.0 ± 26.09^c^	2,121.9 ± 27.83^d^	2,742.8 ± 25.93^a^	2,336.5 ± 30.21^b^	2084.2 ± 52.16^d^	2,314.2 ± 42.51^b^
Average daily gain (g/day)	60.5 ± 8.91^b^	33.6 ± 5.92^d^	72.2 ± 3.62^b^	50.0 ± 1.31^c^	86.2 ± 4.21^a^	57.5 ± 8.93 ^bc^	52.5 ± 9.73^c^	58.1 ± 10.32 ^bc^
Average daily feed intake (g/bird/day)	80.4 ± 2.04^b^	70.6 ± 3.16^c^	95.1 ± 4.82^a^	81.4 ± 3.95^b^	97.32 ± 10.02^a^	85.6 ± 5.89 ^ab^	79.2 ±4.58^b^	83.66 ± 6.42 ^ab^
Feed conversion ratio (kg feed/kg gain)	1.3 ± 0.02^c^	2.1 ± 0.01^a^	1.3 ± 0.00^c^	1.6 ± 0.01^b^	1.1 ± 0.02^d^	1.5 ± 0.08^b^	1.51 ± 0.13^b^	1.44 ± 0.15^b^
Mortality (%)	2.5%	27.5%	2.5%	12.5%	0.0%	7.5%	7.5%	7.5%
Illness (%)	12.5%	42.5%	5.0%	12.5%	0.0%	10.0%	12.5%	5%

^a, b, c^ within the same row that has different superscripts indicate significant differences (p < 0.05); n = 20.

^*^ The time after the start of heat stress exposure. Illness parameters: drooping spirit, diarrhea, and tumbled feathers.

Excessive heat stress treatment alters gut inflammatory gene expression and leads to inflammation. Thus, we evaluated the effect of 1,8-cineole microcapsule on the markers of inflammation. The mRNA levels of NF-қB in CN-HS group were higher than those in C1-HS and C2-HS groups. (Figure 3C). Accordingly, 1,8-cineole microcapsule reduced elevated levels of heat stress induced markers of low-grade inflammation (IL-1β, IL-6, and TNF-α, Figures 3D–F), whereas it significantly elevated levels of the anti-inflammatory cytokine, IL-10 (Figure 3G).

### Dietary 1,8-Cineole Microcapsules Alter Gut Microbiota

Full regions of the 16S rRNA gene were sequenced using microbiome DNA samples obtained from chicken ileum tissues following treatment. After removing sequences less than 200 bp in length, 509,115 sequences of an average length of 1,547 bp were obtained. Clustering analysis generated a total of 3,129 OTUs of high-quality sequences with 97% similarity cutoff.

**FIGURE 4:**
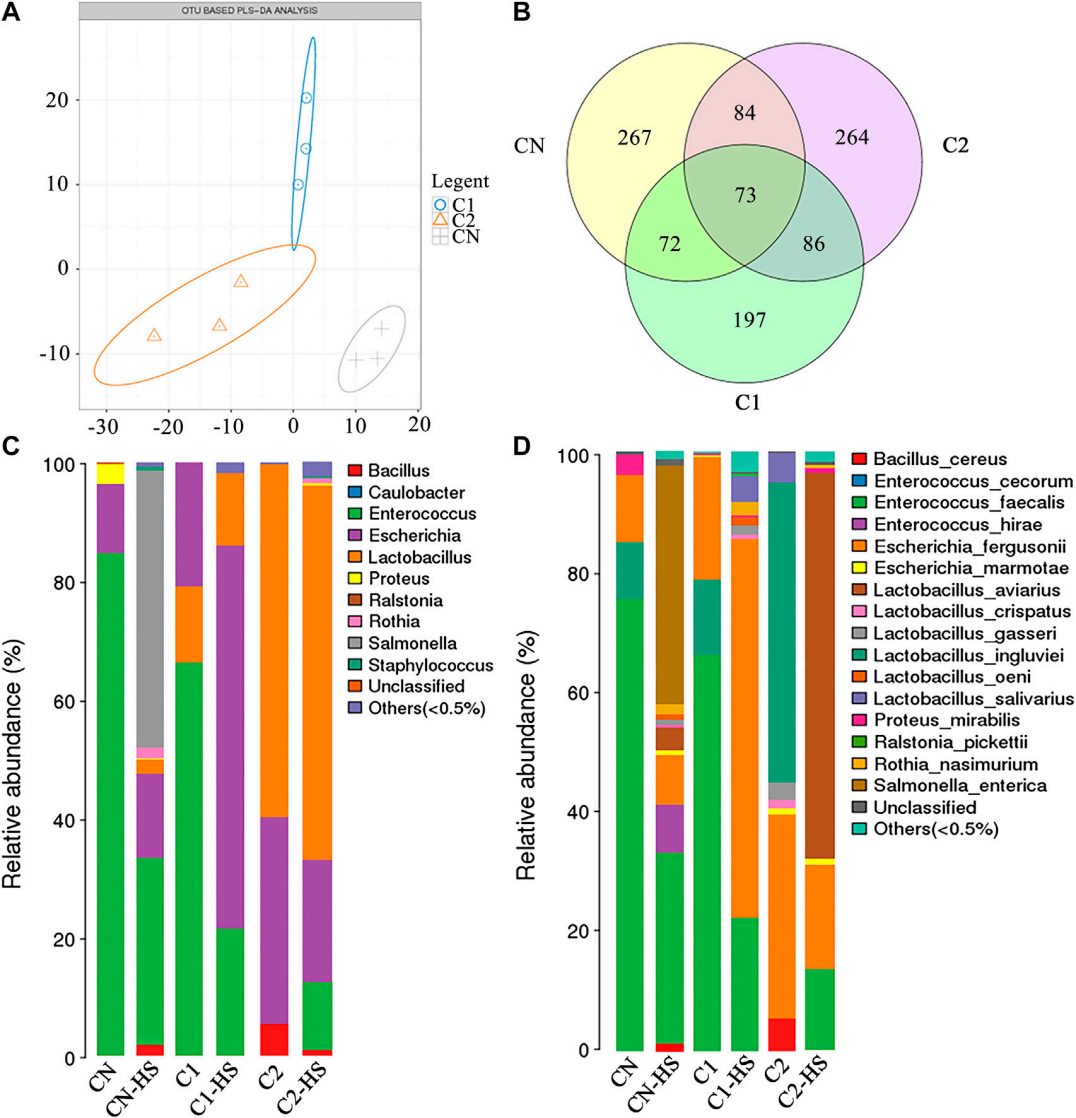
Dietary 1,8-cineole microcapsules alter of gut microbiota. Notes: **(A)** partial least squares discrimination analysis (PLS-DA) based on abundance of OTU. **(B)** Venn diagram for OTU. **(C)** Changes in bacteria genus during the indicated treatment. **(D)** Changes in bacteria species under indicated treatments. n = 6.

Treatment with CN-HS, C1-HS, and C2-HS caused significant difference in gut bacteria abundance based on OTU-based PLS-DA analysis (Figure 4A). A total of 73 species were the same in the treatment groups, whereas 267, 264, and 197 differed among the treatment groups, respectively (Figure 4B). Compared with CN group, C1 and C2 treatments significantly increased the ratio of *Escherichia* and *Lactobacillus*. Compared with CN-HS treatment, C1-HS and C2-HS significantly decreased the ratio of *Salmonella* and *Enterococcus* and increased the ratio of *Escherichia* to *Lactobacillus* (Figures 4C,D).

Further, *in vitro* experiments demonstrated that 1,8-cineole could effectively inhibit the activities of *E. coli*/K88 (isolated from human and chicken and drug-resistant in pig), *S. aureus* MRSA (from human or drug-resistant from human), and *Salmonella* CUCC542 (from human or drug-resistant from human). Notably, *S. aureus* isolated from human had the highest sensitivity to 1,8-cineole, with MIC 0.8 ± 0.04 μL/ml and IZ 18.2 ± 4.32 mm (Table 3).

**TABLE 3:** Inhibition zone and minimum inhibitory concentration of 1,8-cineole against bacteria *in vitro*.

	Source	IZ (mm)	MIC (μl/ml)
*Escherichia coli*	Human	16.3 ± 3.28	2.6 ± 0.05
*E. coli*	Chicken	15.4 ± 4.21	3.4 ± 0.68
*E. coli* (K88)	Drug-resistant from pig	16.3 ± 3.48	2.9 ± 0.32
*Staphylococcus aureus*	Human	18.2 ± 4.32	0.8 ± 0.04
*S. aureus* (MASA)	Drug-resistant from human	13.8 ± 2.69	1.4 ± 0.06
*Bacillus subtilis*	Human	10.6 ± 3.58	3.2 ± 0.17
*Salmonella*	Human	17.4 ± 4.38	1.8 ± 0.06
*Salmonella* (CUCC542)	Drug-resistant from human	15.5 ± 2.53	2.4 ± 0.17

IZ, inhibition zone; MIC, minimum inhibitory concentration; n = 5.

### 1,8-Cineole Microcapsule Protects Gut Microbiota Balance Against Heat Stress

**FIGURE 5:**
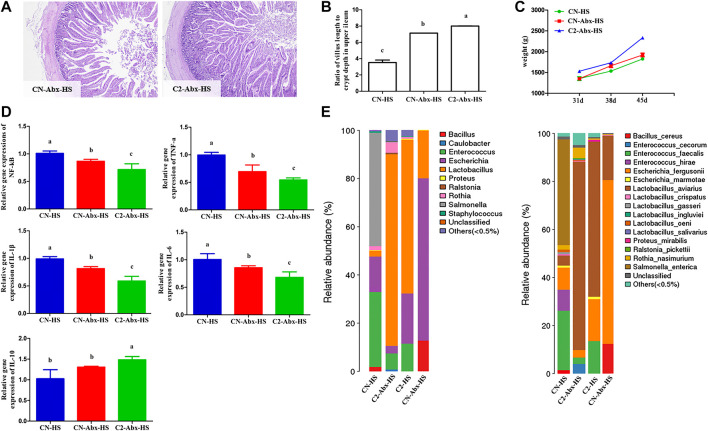
Preventive effects of 1,8-cineole microcapsules on intestinal microbiota mediate heat stress induced weight loss and inflammation. n = 6. Notes: **(A)** the pathological sections of CN-Abx-HS and C2-Abx-HS. **(B)** Ratio of villus length to crypt depth in upper ileum after treatment with heat stress and antibiotic cocktail. **(C)** Weight changes after 31, 38, and 45 d. **(D)** Low-grade inflammation (NF-κB, TNF-α, IL-1, IL-6, and IL-10) expression. **(E)** Changes in bacteria genus and species under CN-HS, CN-Abx-HS, C2-HS, and C2-Abx-HS treatment. Data are expressed as means ± SD, and statistical analysis was performed by ANOVA with a *post hoc Bonferroni’s* multiple-comparison test. ^a, b^ represent significant differences on the same row (*p* < 0.05); n = 6.

Given that heat stress causes imbalanced intestinal microbiota, we next determined whether 1,8-cineole microcapsules can replace the feed antibiotic typically used to protect chickens against heat stress. The various groups were treated with a mixture of broad-spectrum antibiotics to reduce quantitatively microbial populations in the chickens that were subjected to heat stress in the absence or presence of 1,8-cineole microcapsules supplementation (Figure 5A). Interestingly, CN-Abx-HS and C2-Abx-HS groups displayed similar changes in their upper ileum (Figures 5A,B) and presented inflammation (Figure 5D). In contrast with CN-Abx-HS treatment, C2-Abx-HS treatment significantly increased chicken weight (Figure 5C) and *Lactobacillus* spp. (*Lactobacillus_aviarius*) proportion and decreased *Escherichia* spp. (*Escherichia_marmotae*) and *Enterococcus* spp. (*Enterococcus_fergusonii*) proportions (Figure 5E), with decreased proinflammation, such as NF-κB, TNF-α, IL-1β, and IL-6 in the upper ileum.

## Discussion

Stress injury intestinal barrier integrity plays an important role in the pathogenesis of low-grade inflammation in chickens and is a potential factor in weight loss and related health complications (Alhenaky et al., 2017; Xu et al., 2018). To prevent and treat stress induced pathological symptoms, there is a need to find a safe and novel way to limit its development. The present study demonstrates that dietary 1,8-cineole could be utilized in the prevention of high temperature-induced intestinal flora disorders and inflammation. Consequently, we propose a pathway-based mechanism that increased the abundance of *Lactobacillus* by using dietary 1,8-cineole, prevented *Salmonella* expression due to heat stress, and balanced the intestinal microbiota flora and inflammation factors. These changes improved feed conversion ratio, resulting in increased weight.

1,8-Cineole is the main ingredient in an essential oil, which is difficult to preserve for a long time after mixing with feed (Figure 1). Compared with previous results, the 1,8-cineole microcapsules can be used in further study (Asensio et al., 2017). Pharmacokinetics study showed that, compared with 1,8-cineole treatment, 1,8-cineole microcapsules treatment had a longer T_1/2_ and a higher T_max_ and AUC (McLean et al., 2007). The nonencapsulated 1,8-cineole treatment was rapidly absorbed as indicated by a sudden increase in plasma 1,8-cineole with T_max_ of 1 h, which is similar to the T_max_ of 0.67 h reported by Li and colleagues (Li et al., 2018). Encapsulated 1,8-cineole treatment had a slow release with a T_max_ of 4 h. However, the AUC of 1,8-cineole in the encapsulated treatment was higher than that of the nonencapsulated treatment, suggesting that the 1,8-cineole microcapsules treatment can prolong 1,8-cineole absorption time and increase its bioavailability (Table 1).

Our results demonstrated that 1,8-cineole supplementation enhanced *Lactobacillus* growth and decreased *Salmonella* proportion in CN-HS treatment chickens (Figure 4). We also found that 1,8-cineole could effectively inhibit the activities of *Salmonella*, *E. coli*, and *S. aureus* (Table 3). Intestinal flora disturbance can induce inflammation and immune response (Adamczyk-Sowa and Medrek, 2017; Zhang et al., 2018), which in turn can induce intestinal flora. Nonpathogenic intestinal bacteria can promote natural antibodies, which are an important part of nonspecific immune mechanisms and constitute the first line of defense in immune response (Cukrowska et al., 2001). Probiotics contribute to the balance of cytokines and can favorably affect the course of those allergic and inflammatory diseases. The probiotic, *Lactobacillus acidophilus*, reportedly restores the release of proinflammatory cytokines such as TNF-α and IL-1β (Amdekar et al., 2013; Park et al., 2018). *Salmonella* causes acute gut inflammation through the use of its virulence factors, invading the intestinal epithelium and surviving in mucosal macrophages. The inflammatory response enhances the transmission success of *Salmonella enterica* serotype Typhimurium by promoting its outgrowth in the gut lumen (Winter et al., 2010). In our study, C1 and C2 do not significantly affect the proinflammation expressions of these healthy chickens, but C1-HS and C2-HS show a significant effect on the expression of proinflammation compared with CN-HS (Figures 3C-G). These results suggest that 1,8-cineole did not affect inflammation directly but by regulating the microbial flora imbalance to reduce the expression of proinflammation. 1,8-Cineole increased the *Lactobacillus* proportion and decreased the *Salmonella* gut content, which restores the expression of proinflammatory cytokines. Antibiotics mix is typically used to prevent gut bacterial disorders caused by heat stress. Our results showed higher weight gain and proportion of *Lactobacillus* in C2 and lower levels of inflammation in C2-Abx-HS compared to CN-Abx-HS (Figure 5D), suggesting that 1,8-cineole may replace antibiotics for the prevention of heat stress induced inflammation and microbial disorders. Overall, these results suggest that 1,8-cineole microcapsules are beneficial against inflammation, gut microbiota imbalance, and associated weight loss induced by heat stress.

In conclusion, these results prove that interactions among dietary 1,8-cineole microcapsules and inflammation microbiome is a mechanism underlying the growth-promoting effects of 1,8-cineole microcapsules. Considering that intestinal dysbiosis is often associated with inflammatory diseases, the ability to prevent these diseases presents the possibility of 1,8-cineole microcapsules supplementation as an alternative to dietary antibiotics.

## Data Availability Statement

The raw data supporting the conclusions of this article will be made available by the authors, without undue reservation.

## Ethics Statement

The animal study was reviewed and approved by Ethics Committee of the Institute of Modern Biotechnology for the Use of Laboratory Animals.

## Author Contributions

ZJ and KZ designed the study, performed the experiments, analyzed the data, and wrote the paper; ML and WM performed animal studies and analyzed the data. SM and YW designed the study and organized the discussion.

## Funding

This work was supported by Anyang Science and Technology Project, the Key Projects of Universities in Henan (19B180001), Science, Technology Innovation Talents in Universities of Henan Province (18HASTIT035) and National Key R&D Program of China (2017YFD0501003).

## Conflict of Interest

The authors declare that the research was conducted in the absence of any commercial or financial relationships that could be construed as a potential conflict of interest.
